# Salmonella VNP20009-mediated RNA interference of ABCB5 moderated chemoresistance of melanoma stem cell and suppressed tumor growth more potently

**DOI:** 10.18632/oncotarget.7496

**Published:** 2016-02-19

**Authors:** Xiaoxin Zhang, Xiawei Cheng, Yueyang Lai, Yuqiang Zhou, Wenmin Cao, Zi-Chun Hua

**Affiliations:** ^1^ The State Key Laboratory of Pharmaceutical Biotechnology, School of Life Science and School of Stomatology, Affiliated Stomatological Hospital, Nanjing University, Nanjing, 210093, Jiangsu, China; ^2^ Changzhou High-Tech Research Institute of Nanjing University and Jiangsu TargetPharma Laboratories Inc., Changzhou, 213164, Jiangsu, China; ^3^ The State Key Laboratory of Natural Medicines, China Pharmaceutical University, Nanjing, 210009, Jiangsu, China

**Keywords:** bacterial therapy, ABCB5, tumor initiating cells, drug resistance, antitumor

## Abstract

Drug resistance remains an obstacle hindering the success of chemotherapy. Cancer stem cells (CSCs) have been recently found to confer resistance to chemotherapy. Therefore functional markers of CSCs should be discovered and specific therapies targeting these cells should be developed. In our investigation, a small population of B16F10 cells which was positive for ATP-binding cassette sub-family B member 5 (ABCB5) was isolated. This population displayed characteristics similar to those of CSCs and ABCB5 was identified to confer tumor growth and drug resistance in B16F10 cell line. Although targeting ABCB5 by small short interfering RNA delivered by VNP20009 failed to inhibit tumor growth, the combined treatment of VNP-shABCB5 and chemotherapy can act synergistically to delay tumor growth and enhance survival time in a primary B16F10 mice model. Results suggest that the combined treatment of VNP-shABCB5 and chemotherapy can improve the efficacy of chemotherapeutic drugs. Therefore, this combination therapy is of potential significance for melanoma treatment.

## INTRODUCTION

Melanoma is one of the world's most aggressive skin cancer and has become more common nowadays [1]. When melanoma becomes metastatic, it acquires resistance to chemotherapy and tends to disseminate to other organs [2]. At this stage, metastatic melanoma is notorious for its poor prognosis with an average survival time of seven to nine months [3]. Aside from chemotherapeutic drugs such as nitrosoureas, taxanes, vinca alkaloids and platinum-associated drugs, which failed in large random studies, the current chemotherapeutic drugs that theoretically target all metastatic sites, including dacarbazine (DTIC), cyclophosphamide (CTX) and temozolomide, did not significantly increase the overall survival rate of melanoma patients [2, 4]. Therefore, resistance to chemotherapy is a major obstacle in treating metastatic melanoma.

Increasing evidence has shown that a small proportion of cells in melanoma may contribute to therapy resistance [5, 6]. In contrast to other tumor cells, these cells which contain clonal long-term repopulation and self-renewal capacity are known as cancer stem cells (CSCs) and at the apex of tumor hierarchy [7–9]. Moreover CSCs have been frequently reported to correlate with neoplastic progression, metastatic potential and poor prognosis in melanoma patients [10–12]. CSCs are thought to be resistant to chemotherapy because of various reasons such as their quiescent or slowly proliferating nature, high expression level of ATP-binding cassette (Abc) drug pumps, intrinsically high levels of anti-apoptotic molecules, relative resistance to oxidative or DNA damage, and efficiency of DNA repair [13]. In particular, ATP-binding cassette sub-family B member 5 (ABCB5), the mediator of chemoresistance, is overexpressed by CSCs in diverse human malignancies, including melanoma stem cells. ABCB5 has also been identified as a CSC marker in human melanoma [10, 12, 14–18]. The expression of ABCB5 strongly overlapped with clinical tumor progression, therapeutic resistance, and recurrence in malignant melanoma [3, 10, 17, 19–23]. Furthermore, ABCB5 is frequently correlated with the *in vitro* clonogenic potential of melanoma cells [24–26]. Previous research also reported that blocking ABCB5 can inhibit tumor growth and reverse the resistance of CSCs to chemotherapeutic agents [10, 27]. Therefore, identifying and targeting CSCs can significantly improve cancer therapies.

In this study, we treated tumor-bearing mice with the engineered VNP20009, which is a variant of *Salmonella* and has been proven safe in phase I clinical trial, together with CTX. We found this combined treatment of VNP20009 carrying shABCB5 with CTX efficiently reduced tumor growth and prolonged survival time by reducing ABCB5 expression and inhibiting chemotherapy resistance.

## RESULTS

### Identification of potential CSC surface markers of murine melanoma cells

**Figure 1:**
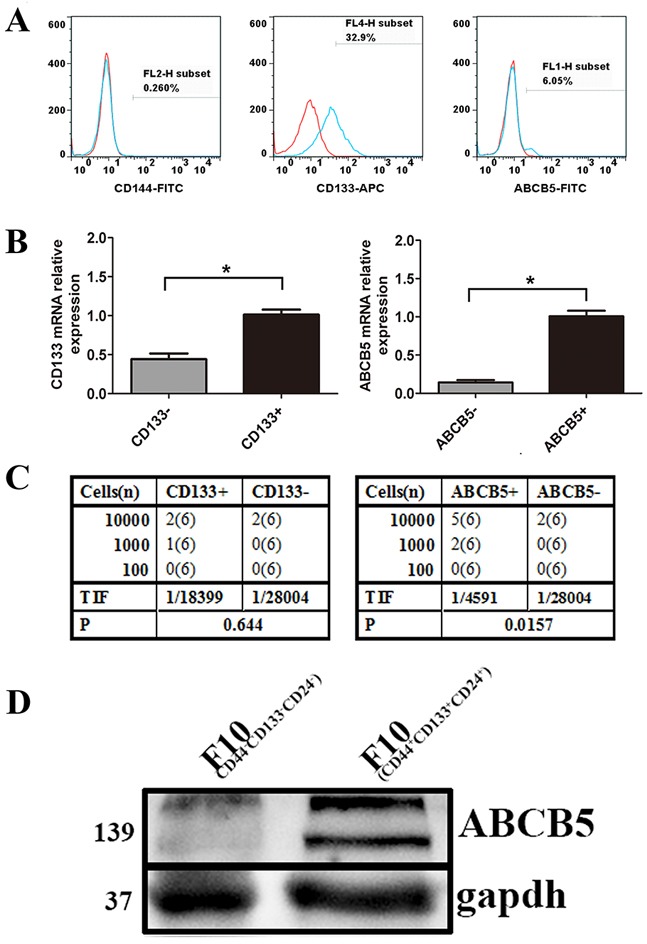
Identification of potential CSC marker of murine melanoma cells **A.** The expression of CD133, ABCB5 and CD144 on the cells obtained by three rounds of selection. **B.** mRNA levels of subpopulation stained with CD133 and ABCB5. Data are presented as mean ± SD. *P < 0.05 for CD133+ versus CD133− or ABCB5+ versus ABCB5−. **C.** Limiting dilution assay showing tumor initiating ability of CD133+, CD133−, ABCB5+, ABCB5− *in vivo*. A series number of cells were inoculated into six mice per group. Tumor development was recorded daily. TIF was calculated using the ELDA software. **D.** Western blots for ABCB5 expression by CD44+CD133+CD24+ versus CD44−CD133−CD24− melanoma cell (gapdh-loading control).

To enrich CSCs, B16F10 cells were subcutaneously (s.c.) inoculated into the mid-right flank of C57/B6 mice. When the tumor size reached 500 mm^3^, the mice were sacrificed. As mentioned in the *Materials and methods*, the treated tumor cells were re-inoculated into the C57/B6 mice. After three rounds of selection, we probed the cells using antibodies against potential CSC markers, including CD144, CD133 and ABCB5 [28]. Although no cells expressed CD144, a subpopulation of the cells expressing CD133 (32.9%) or ABCB5 (6.05%) was detected (Figure 1A). We then investigated the tumorigenicity of the cells expressing CD133 or ABCB5. After three rounds of selection, we stained the cells using antibody against CD133 or ABCB5. CD133-positive, CD133-negative, ABCB5-positive and ABCB5-negative cells were sorted by FACSAria™ III. Protein expression of the individual population was confirmed by real-time PCR, which showed that the positive groups expressed higher levels of CD133 or ABCB5 than the negative groups (Figure 1B).

A rigorous and functional test for CSCs is the limiting dilution assay. Accordingly, CD133 positive or negative cells were injected into the mice, but we did not observe any differences with respect to tumorigenicity. The tumorigenic cell frequency for the CD133-positive population was 1/18399 (1/5709-1/59298), whereas that for the CD133-negative population was 1/28004 (1/7039-1/111406) (Figure 1C). No significant difference was detected between the two groups of tumorigenic cell frequency. However, cells from the ABCB5-negative population exhibited a six fold reduction of tumor-initiation frequency. The tumorigenic cell frequency for the ABCB5-positive was 1/4591 (1/1900–1/10993), whereas that for the ABCB5-negative population was 1/28004 (1/7039-1/111406) (Figure 1C).

To further prove the potential of ABCB5 as a CSC marker in murine melanoma cells, we sorted B16F10 (CD44+CD133+CD24+) by FACSAria™ III which have been proved to have biological characteristics of CSC. We then analyzed the expression of ABCB5 in B16F10 (CD44+CD133+CD24+) and B16F10 (CD44-CD133-CD24-). Western blot analysis showed a higher expression of ABCB5 in B16F10 (CD44+CD133+CD24+), with characteristics of CSCs, than in B16F10 (CD44-CD133-CD24-) (Figure 1D). Overall, these data suggest ABCB5 as a CSC marker of murine melanoma.

### ABCB5 regulates murine melanoma growth and multidrug resistance

**Figure 2:**
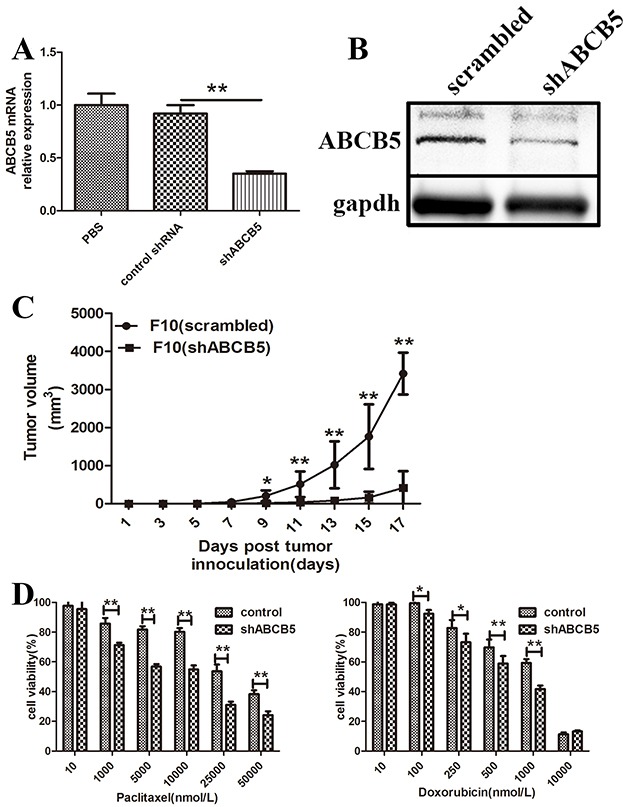
ABCB5 regulates murine melanoma growth and drug resistance **A.** mRNA levels of ABCB5 from B16F10 lysates following transfection of shRNA constructs. Data are presented as mean ± SD. **P < 0.01 for control shRNA versus shABCB5. **B.** Western blot for ABCB5 expression by B16F10 (scrambled) vs. B16F10 (shABCB5). **C.** The tumor growth curve of B16F10 (scrambled) and B16F10 (shABCB5) (*n* = 6 each). Data are presented as mean ± SD. **P < 0.01 for B16F10 (scrambled) versus B16F10 (shABCB5). **D.** Drug dependent cell killing of B16F10 (shABCB5) versus B16F10 (scrambled).*for P < 0.05, ** for P < 0.01.

To elucidate the functional role of ABCB5 in murine melanoma growth, we silenced ABCB5 in B16F10 by shRNA. Western blot and qPCR analyses confirmed that shABCB5 can silence the level of ABCB5 (Figure 2A, 2B). Afterward, we inoculated 10,000 cells of B16F10 (shABCB5) or respective control into the mid-right flank of C57B6 mice. Tumor formation and volume were recorded every other day. Tumor growth upon ABCB5 inhibition was severely impaired throughout the course of the experiment. The tumor volumes of mice inoculated with B16F10 (shABCB5) significantly decreased compared with the respective controls, demonstrating that ABCB5 performs a functional role in murine melanoma growth (Figure 2C).

B16F10 (shABCB5) and its respective control were treated with varying concentrations of paclitaxel or doxorubicin at indicated times to analyze the functional role of ABCB5 in multidrug resistance by MTT. Paclitaxel and doxorubicin have been reported to be closely related to drug resistance and ABCB5 expression in melanoma. MTT assay showed that the viability of B16F10 (shABCB5) decreased to 71%, 56%, 54%, 31% and 24% after treatment with 1, 5, 25 and 50 μM paclitaxel respectively, whereas the viability of the control group was 85%, 81%, 80% 53% and 38% correspondingly (Figure 2D). The viability of B16F10 (shABCB5) treated with doxorubicin also significantly decreased (73% vs. 82% for 0.25 μM, 58% vs. 69% for 0.5 μM and 41% vs. 59% for 1 μM) (Figure 2D). Thus inhibition of ABCB5 can reverse the resistance of B16F10 melanoma cells to paclitaxel and doxorubicin. These results demonstrated two functional roles of ABCB5 in murine melanoma growth and multidrug resistance, suggesting that ABCB5 is a promising target for melanoma therapy.

### Construction and anticancer ability of VNP-shABCB5

**Figure 3:**
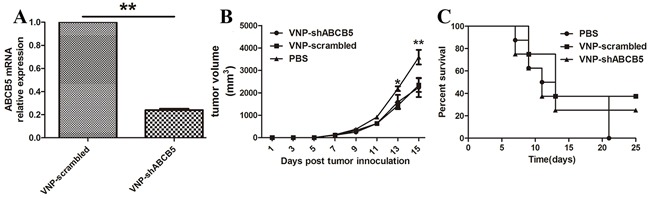
Targeted silencing of ABCB5 did not significantly suppress tumor growth **A.** Silencing of ABCB5 expression in B16F10 tumor following i.p. injection of VNP-shABCB5. Data are presented as mean ± SD. **P < 0.01 for VNP-scrambled versus VNP-shABCB5. **B.** Tumor growth curves. The tumor-bearing mice were injected i.p. with PBS, VNP-scrambled and VNP-shABCB5 (*n* = 8, each group). Tumor volumes among different groups were compared. Data are presented as mean ± SD. *P < 0.05 for PBS versus VNP-scrambled and VNP-shABCB5. **C.** Kaplan-Meier survival curves of the mice bearing B16F10 melanomas. Data were analyzed by the log-rank test. * for P < 0.05 for VNP-shABCB5 versus VNP-scrambled.

Considering that ABCB5 is an attractive therapeutic target, we tend to silence the expression of ABCB5 carried by VNP20009, which has been reported to specifically target CSC-like population and reduce its growth [29, 30]. After generating VNP20009 carrying shABCB5, we monitored whether systemic delivery of *Salmonella* can effectively silence the expression of ABCB5 *in vivo*. Tumor-bearing mice were randomly divided into three groups and treated with PBS, VNP-scrambled and VNP-shABCB5, respectively. As shown in Figure 3B and 3C, there was no significant attenuation of tumor growth in the mice treated with VNP-shABCB5 compared with VNP-scrambled and the survival time of the mice between the two groups was not significantly different. All the mice were sacrificed and each tumor was homogenized. Total RNA was isolated for RT-PCR. Our results showed that the expression of ABCB5 significantly decreased in the mice treated with VNP-shABCB5 five days post injection (Figure 3A). Taken together, these findings confirmed that VNP-shABCB5 succeeded in silencing ABCB5 mRNA but failed to improve the anticancer ability compared with VNP-scrambled.

### Combined therapy of VNP-shABCB5 and CTX suppress tumor growth

**Figure 4:**
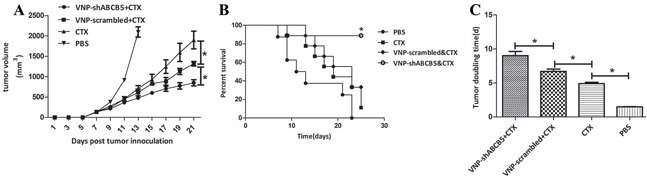
The combined therapy of VNP-shABCB5 with CTX delayed tumor growth and enhanced survival time in B16F10 mice model **A.** Tumor growth curves. Tumor-bearing mice were injected i.p. with PBS, CTX, VNP-scrambled plus CTX, VNP-shABCB5 plus CTX (*n* = 9, each group). Tumor volumes among different groups were compared. Data are presented as mean ± SD. *, P < 0.05. **B.** Kaplan-Meier survival curves of mice bearing B16F10 melanomas. Data were analyzed by the log-rank test. *, P < 0.05. **C.** Tumor doubling time. Data are presented as mean ± SD. *, P < 0.05.

Given that ABCB5 mediated drug resistance in B16F10, we delivered VNP-shABCB5 together with CTX to moderate drug resistance in chemotherapy. When the tumor was palpable, 40 mg/kg CTX was administrated i.p. every other day, as described in Jia's work [31]. 1×10^5^ CFU of VNP-shABCB5 or VNP-scrambled was simultaneously administered at the beginning of CTX treatment. Although the treatment of VNP-scrambled together with CTX markedly enhanced the anticancer ability, the combined therapy of VNP-shABCB5 and CTX further attenuated tumor growth and prolonged the survival time compared with the other groups (Figure 4). The tumor volume in VNP-shABCB5 plus CTX group significantly decreased compared with the other groups (P<0.05) (Figure 4A). Kaplan Meier survival assay showed that the survival rate of mice in the VNP-shABCB5 plus CTX group significantly increased compared with those in the PBS, CTX and VNP-scrambled plus CTX groups (log-rank tests, P<0.05) (Figure 4B). The tumor doubling time was significantly prolonged from 1.49 d (CI, 1.42 d to 1.56 d) in the PBS control group, 4.89 d (CI, 4.55 d to 5.28 d) in the CTX group, or 6.68 d (CI, 6.15 d to 7.30 d) in the VNP-scrambled plus CTX group to 8.94 d (CI, 10.13 d to 7.99 d) in the VNP-shABCB5 plus CTX group (P<0.05) (Figure 4C).

**Figure 5:**
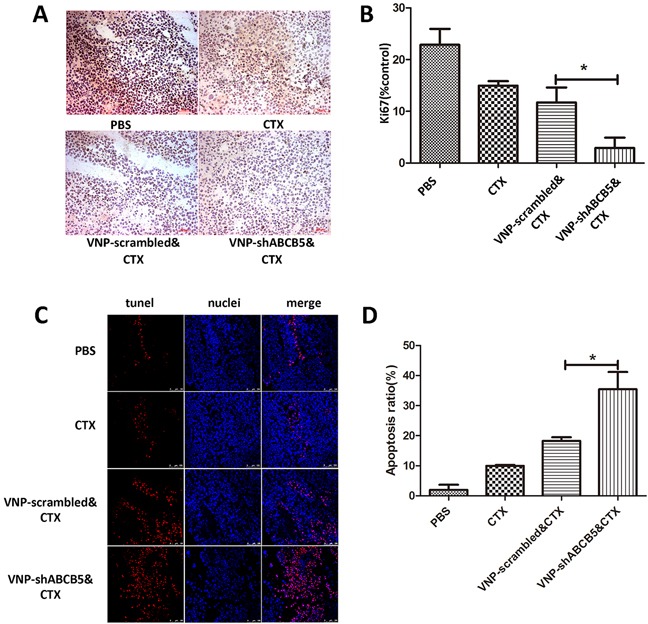
The combined therapy of VNP-shABCB5 with CTX promotes cell death and inhibits cell proliferation **A.** Immunohistochemistry for Ki-67 (stained brown) revealed the expression of Ki67 in the tumors of mice treated with PBS, CTX, VNP-scrambled plus CTX, VNP-shABCB5 plus CTX. **B.** Quantification of Ki-67 expression. Data are presented as mean ± SD. *P < 0.05 for VNP-scrambled & CTX versus VNP-shABCB5 & CTX. **C.** TUNEL staining showed the death of B16F10 melanoma cells from the mice treated with PBS, CTX, VNP-scrambled plus CTX, VNP-shABCB5 plus CTX. **D.** Quantitative analysis of TUNEL-positive cells. Data are presented as mean ± SD. *P < 0.05 for VNP-scrambled & CTX versus VNP-shABCB5 & CTX.

To determine whether the synergistic effect of VNP-shABCB5 and CTX on tumor growth was caused by moderating the chemotherapy resistance, we stained the tumor sections with Ki-67 antibody for CTX, which acted by interfering DNA replication and inhibiting tumor growth. Immunochemical analysis of the tumor with Ki-67 antibody revealed that although CTX treatment alone could attenuate Ki-67 expression, the percentage of Ki-67 positive staining significantly decreased in the combined therapy of CTX with VNP-shABCB5 (Figure 5A, 5B). In addition, we stained the tumor sections with an *in situ* TUNEL assay. As shown in Figure 5C and 5D, TUNEL-positive staining was observed in nearly half the area of the section, indicating that more cell death occurred due to the combined therapy. Consistent with previous reports, the combined therapy of VNP-scrambled and CTX can also promote cell death to a lesser extent. CTX alone also showed a significantly less effective induction of death in melanoma cells *in vivo* than the combined therapy of either VNP-shABCB5 plus CTX or VNP-scrambled vector plus CTX.

## DISCUSSION

Chemotherapy resistance is a major factor impairing melanoma treatment. Because of intratumoral heterogeneity, chemotherapy only kills cells that are sensitive to drugs and leaves the drug-resistant cells unharmed. Indeed, mounting evidence has shown that CSCs play an important role in chemotherapy resistance [32, 33].

The present study aimed to design a novel therapy targeting CSCs in B16F10 cells to improve the efficacy of chemotherapy. Both CD133 and ABCB5 have been identified as CSC markers of human melanoma. CD133-positive cells have been reported to display multi-potent differentiation *in vitro* and tumorigenic growth *in vivo* [34]. CD133 down-regulation in melanoma cells has led to the decrease in proliferation rate, cellular migration and inhibition of melanosphere formation; such findings confirmed a key role for CD133 in melanoma tumor formation [35, 36]. However in the present study we did not observe any difference in tumor initiation capacity when comparing CD133+ with CD133- groups, suggesting that CD133 alone cannot identify CSCs in B16F10. These findings are consistent with Jun Dou's work. However, ABCB5-positive cells exhibited higher tumorigenicity in C57/B6 mice than cells not expressing the protein. More importantly, a higher expression of ABCB5 was found in B16F10 (CD44+CD133+CD24+), which have characteristics of CSC, than in B16F10 (CD44-CD133-CD24-) [37]. Furthermore inhibition of ABCB5 can impair tumor growth *in vivo* and reverse the resistance of B16F10 melanoma cells to chemotherapeutic drugs *in vitro*. Although ABCB5 has been extensively studied in diverse human malignancies, few investigated ABCB5 in murine melanoma. Our results provide initial evidence that ABCB5 is not only a marker of murine melanoma CSCs, but also perform functional roles in murine melanoma growth and multidrug resistance. This implicates that ABCB5 serves as a key driver of murine melanoma aggressiveness, but more work need to be done to elucidate the mechanism.

Considering that ABCB5 is a promising target for melanoma therapy, we selected VNP20009 carrying shRNA against ABCB5 to inhibit the melanoma growth in mice. VNP20009 was developed by depleting msbB and pur I in the background *Salmonella* 14028 and has been proven safe in the phase I clinical trial [38, 39]. Since VNP20009 preferentially accumulates in tumor at a ratio greater than 1000/1 compared with normal organs, it is widely used as a delivery vehicle expressing exogenous proteins such as TNF-related *apoptosis*-inducing ligand (*TRAIL*), Histidine-proline-rich glycoprotein (HPRG), or carrying shRNA-expressing plasmid to silence the expression of genes like STAT3 and indoleamine 2,3-dioxygenase (IDO) [40–43]. Recent evidence showed that *Salmonella* can predominantly reside in the CSC region of tumor and is a promising therapy for chemo-resistant pancreatic cancer stem-like cells [29, 30]. In our study when the tumor was palpable, VNP20009 carrying shABCB5 was administered into the tumor-bearing mice and its anticancer capacity was recorded. Our results showed that the expression of ABCB5 was decreased in the tumor of mice treated with VNP-shABCB5, which indicated that VNP-mediated shRNA could specifically target ABCB5 and silence its expression. Although both VNP-scrambled and VNP-shABCB5 effectively inhibited tumor growth, there was no significant difference between the tumor volumes of the mice treated with VNP-scrambled and VNP-shABCB5. This was not surprising. In contrast to published reports, in which ABCB5 has been silenced on the day of tumor implantation, we began the treatment when the tumor was already palpable. Given that tumor contain not only tumor-initiating cells but also proliferating progenitor-like cells, all cancer cells that may potentially contribute to the disease should be killed. Therefore, VNP-shABCB5 should be combined with chemotherapeutic drugs.

Our results were promising. The combined therapy of VNP-shABCB5 and CTX significantly reduced tumor growth and prolonged the survival time compared with those of the VNP-scrambled plus CTX, CTX, and PBS groups. The combined therapy significantly induced tumor cell apoptosis and inhibited its proliferation. VNP-scrambled could also improve the anticancer ability of CTX, which was consistent with Jia's work [31]. Two mechanisms may contribute to the synergistic antitumor effects of CTX and VNP-shABCB5. First, ABCB5 blockage mediated by VNP20009 may render cells more sensitive to chemotherapy agents, therefore CTX kills CSCs as well as other tumor cells. Secondly, *Salmonella* can play a direct role in the CSC population and inhibit CSCs growth *in vivo*. This may further explain Jia's work in which VNP20009 improved cyclophosphamide chemotherapy at maximum tolerated dose and low-dose metronomic regimens in a murine melanoma model by targeting and killing CSCs in B16F10 melanoma cells.

In summary, we found a potential CSC marker of B16F10 melanoma and designed an approach of combining VNP20009 carrying shRNA targeting against CSC marker with chemotherapy. The combined therapy can inhibit tumor growth and prolong survival time by moderating chemotherapy resistance. We believe that this strategy opens new windows in melanoma treatment and potentiates future researches and applications of combinatorial therapies.

## MATERIALS AND METHODS

### Bacteria, cell lines and animals

Lipid A modified (msbB^−^), auxotrophic (purI^−^) *Salmonella typhimurium* VNP20009 were obtained from American Type Culture Collection (ATCC, USA) and cultured in modified Luria–Bertani (LB) media. The B16F10 cells were obtained from ATCC and cultured in 5% CO_2_ in a humidified atmosphere in *Dulbecco's Modified Eagle's medium* (*DMEM*) with 10% fetal bovine serum (FBS). Six-week-old female C57/B6 mice were purchased from the Comparative Medicine Center of Yangzhou University and maintained in pathogen-free conditions for one week before the start of the experiment.

### Flow cytometry

Before sorting, B16F10 cells were suspended in ice-cold PBS to a final density of 10^6^/ml and injected into mid right flank of C57/B6 mice with 100 μl. When tumor grew up, the mice with tumor were then sacrificed, and the isolated cells from the tumor tissue were cultured. These cells were re-inoculated into C57B6 mice after *in vitro* culture. After three times of the repeated inoculation approach, the cells were stained with antibody against CD144 (FITC-), CD133 (APC-), ABCB5 (FITC-) at 4°C for 30 min. The cells were analyzed on a FACSCalibur using CELLQUEST software (BD Biosciences).

### Limiting dilution transplantation assay

For limiting dilution assay, 100, 1000, 10,000 cells of the individual subpopulation (CD133+, CD133-, ABCB5+ and ABCB5-), which was generated using anti-CD133 and anti-ABCB5 antibody labeling and sorted by FACSAria™ III, were subcutaneously transplanted into mid-right flank of C57/B6 mice. Tumor development was then monitored on a daily basis. Tumor-initiation frequency (TIF) was calculated by the ELDA Software [44].

### Cell viability assay

10^3^−10^4^ cells were seeded triplicate in 96-well plates. After 12 hours incubation, B16F10 (scrambled) and B16F10 (shABCB5) were treated with various concentrations of paclitaxel or doxorubicin for 24 hours. Cell viability was determined by MTT assay as described previously [45]. In brief, after drug treatment, MTT (5 mg/ml, 100 μl/well) was added to each well and after additional 4 hours incubation, the supernatant was removed and 100 μl/well DMSO was added. After shaking the plates for 10 min to dissolve the formazan, absorbance of each well was recorded with a microplate reader (Safire, TECAN, Switzerland) at 570 nm.

### Real time quantitative PCR assay

Total RNA was isolated with TRIzol reagent (Invitrogen). cDNA was generalized using ReverTra Ace® qPCR RT Kit (Toyobo). Real-time quantitative PCR was done with primers as follows to determine the expression level of CD133 and ABCB5.

β-actin sense, 5′-GAGACCTTCAACACCCCAGC-3′

β-actin antisense, 5′-ATGTCACGCACGATTT CCC-3′

CD133 sense, 5′-GAAAAGTTGCTCTGCGAACC-3′

CD133 antisense, 5′-TCTCAAGCTGAAAAGCA GCA-3′

ABCB5 sense, 5′-GTGGCTGAAGAAGCCTT GTC-3′

ABCB5 antisense, 5′-TGAAGCCGTAGCCCTCT TTA-3′

Real-time quantitative PCR was performed on StepOne Real-Time PCR System (Applied Biosystems, USA) with AceQ^®^ qPCR SYBR^®^ Green Master Mix (Vazyme China). Data was analyzed by StepOne Software 2.1 (Applied Biosystems, USA) according to the manufacturer's specifications. β-actin was used as a control.

### shRNA plasmid construction and bacterial transformation

shRNA against ABCB5 was tested for silencing by transformation of B16F10 cells followed by RT-PCR. The pRNA U6.1 vector containing the 19-mer shRNA sense sequence GGTCGATGAACAAATGGAA silenced more than 60% of ABCB5. The pRNA U6.1 vector and the one containing the shRNA sequence were respectively electroporated into VNP20009 by Gene Pulser Xcell (Bio Rad) at 2.5 kV, 25 mF and 400Ω.

### Tumor implantation, CTX and bacterial treatment

1×10^5^ B16F10 cells were subcutaneously (s.c.) inoculated into the mid-right flank of the C57B6 mice. On the seventh day when the tumor was palpable, the tumor-bearing mice were randomly divided into five groups: PBS, VNP-scrambled, VNP-shABCB5, VNP-scrambled&CTX, VNP-shABCB5&CTX. Mice in the PBS group were intraperitoneally injected with 100 μl PBS. Mice in the VNP-scrambled or VNP-shABCB5 groups were injected with 1×10^5^ cfu VNP-scrambled or VNP-shABCB5. Mice in the VNP-scrambled&CTX or VNP-shABCB5&CTX group were intraperitoneally injected with 45 mg/kg CTX as well as 1×10^5^ cfu VNP-scrambled or VNP-shABCB5. Once the bacteria were inoculated into the tumor-bearing mice, the length and width of the tumor were measured every two days using a Vernier caliper (Mytutoyo Co., Japan) across its two perpendicular diameters. Tumor volume was calculated using the following equation: tumor volume = length × width^2^ × 0.52. The number of dead mice was recorded to evaluate the survival rate.

### Immunohistochemistry and terminal deoxynucleotidyl transferase dUTP nick end labeling (TUNEL) assay

Five days after treatment, mice in the PBS, VNP-scrambled & CTX, VNP-shABCB5 & CTX group were sacrificed. After freezing in isopentane pre-cooled with liquid nitrogen, the OCT-embedded tumor was cut into 10 μm sections. The tumor frozen sections were incubated with rabbit polyclonal antibody against Ki67 to analyze the expression of Ki67. Apoptotic cells *in situ* were determined by TUNEL Bright Red Apoptosis Detection Kit (Vazyme).

### Statistics analysis

The results were analyzed with GraphPad Prism and presented as mean ± SD. One-way ANOVA was used to calculate the statistics difference among groups. A value of p<0.05 indicates statistical difference.
